# Leaded aviation gasoline exposure risk and child blood lead levels

**DOI:** 10.1093/pnasnexus/pgac285

**Published:** 2023-01-10

**Authors:** Sammy Zahran, Christopher Keyes, Bruce Lanphear

**Affiliations:** Department of Economics, Colorado State University, Fort Collins, CO 80523, USA; Department of Epidemiology, Colorado School of Public Health, Aurora, CO 80045, USA; Mountain Data Group, Fort Collins, CO 80524, USA; Department of Economics, Colorado State University, Fort Collins, CO 80523, USA; Mountain Data Group, Fort Collins, CO 80524, USA; Faculty of Health Sciences, Simon Fraser University, Burnaby, BC V5A 1S6, Canada

**Keywords:** aviation gasoline, child blood lead, piston-engine aircraft

## Abstract

Lead-formulated aviation gasoline (avgas) is the primary source of lead emissions in the United States today, consumed by over 170,000 piston-engine aircraft (PEA). The U.S. Environmental Protection Agency (EPA) estimates that four million people reside within 500m of a PEA-servicing airport. The disposition of avgas around such airports may be an independent source of child lead exposure. We analyze over 14,000 blood lead samples of children (≤5 y of age) residing near one such airport—Reid-Hillview Airport (RHV) in Santa Clara County, California. Across an ensemble of tests, we find that the blood lead levels (BLLs) of sampled children increase in proximity to RHV, are higher among children east and predominantly downwind of the airport, and increase with the volume of PEA traffic and quantities of avgas sold at the airport. The BLLs of airport-proximate children are especially responsive to an increase in PEA traffic, increasing by about 0.72 μg/dL under periods of maximum PEA traffic. We also observe a significant reduction in child BLLs from a series of pandemic-related interventions in Santa Clara County that contracted PEA traffic at the airport. Finally, we find that children’s BLLs increase with measured concentrations of atmospheric lead at the airport. In support of the scientific adjudication of the EPAs recently announced endangerment finding, this in-depth case study indicates that the deposition of avgas significantly elevates the BLLs of at-risk children.

Significance Statement In the United States, hundreds of millions of gallons of tetraethyl lead-formulated gasoline are consumed by piston-engine aircraft (PEA) annually, resulting in an estimated half-million pounds of lead emitted into the environment. About four million persons reside, and about six hundred K-12th grade schools are located, within 500 meters of PEA-servicing airports. In January 2022, the US Environmental Protection Agency launched a formal evaluation of “whether emissions of lead from PEA cause or contribute to air pollution that endangers public health or welfare.” In support of the EPA’s draft endangerment finding and request of public comment, an ensemble of evidence is presented indicating that the deposition of leaded aviation gasoline significantly elevates the blood lead levels of at-risk children.

## Introduction

Over the last four decades, the blood lead levels (BLLs) of children in the United States declined significantly, coincident with a series of policies that removed lead from paint, plumbing, food cans, and automotive gasoline. Most effective among these interventions was the phase-out of tetraethyl lead (TEL) from automotive gasoline under provisions of the Clean Air Act of 1970 and amendments in 1990.

While TEL is no longer used as an additive in automotive gasoline, it remains a constituent in aviation gasoline (avgas) used by an estimated 170,000 piston-engine aircraft (PEA) nationwide. TEL is one of the best-known additives for mitigating the risk of engine knocking or detonation, which can lead to sudden engine failure. In the United States, hundreds of millions of gallons of TEL-formulated gasoline are consumed by PEA annually, resulting in an estimated half-million pounds of lead emitted into the environment. Today, the use of lead-formulated avgas accounts for about half to two thirds of current lead emissions in the United States (1). In a recently published consensus study on options for reducing lead emissions by PEA by the National Academies of Sciences, Engineering, and Medicine, the authors note: “While the elimination of lead pollution has been a U.S. public policy goal for decades, the GA [General Aviation] sector continues to be a major source of lead emissions” (2).

Several studies have linked avgas use to elevated atmospheric lead levels in the vicinity of airports (3–8). The U.S. EPA estimates that four million persons reside, and about six hundred K-12th grade schools are located within 500 meters of PEA-servicing airports (9). Two studies have statistically linked avgas use to BLLs of children residing in the vicinity of general aviation airports. In their groundbreaking study, Miranda et al (10) reported a striking relationship between child BLLs and airport proximity, noting that “[t]he estimated effect on BLLs exhibited a monotonically decreasing dose-response pattern” with children at 500 and 1,000 meters of an airport at greatest risk of elevated BLLs. In a study involving over 1 million children and 448 airports in Michigan, Zahran et al (11) found that child BLLs: (1) increased dose-responsively in proximity to airports; (2) declined measurably among children sampled in the months after the tragic events of 9-11, resulting from an exogenous reduction in PEA traffic; (3) increased dose-responsively in the flow of PEA traffic across a subset of airports; and (4) increased in the percent of prevailing wind days drifting in the direction of a child’s residence.

On the basis of such studies and decades of research on the harm to human health caused by lead, various public interest organizations have petitioned the EPA to make an endangerment finding under Section 231 of the Clean Air Act for aviation gasoline (avgas) emissions. While the EPA recognizes that there is no known safe level of lead exposure, it has cautioned that additional scientific research is needed “to differentiate aircraft lead emissions from other sources of ambient air lead” (12) that may cause elevated BLLs in nearby children.

Subsequent to a report prepared for the County of Santa Clara showing that exposure to leaded avgas contributes to child BLLs (13), and a new petition by various nonprofit and governmental organizations, in January 2022 the EPA launched a formal evaluation of “whether emissions of lead from PEA cause or contribute to air pollution that endangers public health or welfare.” In recent weeks, the EPA published its draft endangerment finding and is currently accepting public comment. In this paper, we present relevant information for the scientific adjudication of the EPA’s draft endangerment finding, supporting the conclusion that emissions from PEA independently contribute to child BLLs, potentially endangering the health and welfare of populations residing near over 21,000 general aviation airports that service avgas-consuming aircraft.

Our paper analyzes the BLLs of children (≤5 y of age) over a 10-y observation period (from 2011 January 31 to 2020 December 31) who reside near one PEA-servcing airport–Reid-Hillview Airport (RHV) in Santa Clara County. Of the more than 21,000 airports appearing in the 2017 EPA National Emissions Inventory, RHV ranks 36th in terms of the quantity of emissions released. From 2011 January to 2018 December , 2.3 million gallons of avgas were sold at RHV. At about 2 grams of lead per gallon, and based on an EPA estimate that 95% of lead consumed is emitted in exhaust, over this 8-y period about five metric tons of lead was emitted at RHV.

The purpose of our analysis is to test key indicators of exposure risk, including child residential distance, residential near angle (or downwind residence), and volume of traffic from the date of the blood draw. We follow with extended analyses involving the statistical interaction of residential distance and air traffic, a natural experiment exploiting an observed contraction in PEA traffic at RHV following pandemic-related social distancing measures enacted countywide, and an analysis linking child BLLs to atmospheric lead measurements at the airport. Across all tests, we find consistent evidence that exposure to avgas increases child BLLs, adding a data-rich and in-depth case study to the nascent scientific literature on the epidemiological hazard of leaded avgas.

## Results

### Main analysis

We begin with analysis of our three main indicators of avgas exposure risk: (1) child residential distance, (2) child residential near angle, and (3) child exposure to PEA traffic. Table 1 reports regression coefficients on our main indicators of exposure risk. Our response variable of child BLL is measured in μg/dL units. Following others (10,11), residential distance is also divided into intervals: <0.5 miles (or <0.8 km), 0.5 to 1 mile (or 0.8 to 1.6 km), and 1 to 1.5 miles (or 1.6 to 2.4 km) from RHV (Our inner orbit of exposure risk at < 0.5 miles conforms to previous research. Miranda et al (10) find that children at 500m to 1km from a general aviation airport in North Carolina are at highest at-risk of presenting with elevated BLLs. Zahran et al (11) find that sampled children within 1km of 448 airports in Michigan are at greatest risk. The EPA (14) maintains that children within 500m of PEA-servicing airports are at highest risk of exposure to aviation-related atmospheric lead. Our inner distance of <0.5 miles sits between the consensus range of exposure risk at 500m to 1km).

**Table 1.** Coefficients of residential distance, near angle, and PEA Traffic vis-à-vis Child BLLs.

BLLs (μg/dL)	(1)	(2)	(3)	(4)^†^
1. Distance RHV (0.5 to 1 miles)	−0.161**	−0.231***	−0.234***	−0.055***
	(0.070)	(0.082)	(0.067)	(0.018)
2. Distance RHV (1 to1.5 miles)	−0.173***	−0.233***	−0.235***	−0.058***
	(0.065)	(0.080)	(0.066)	(0.018)
3. PEA traffic volume	0.312***	0.319***	0.168**	0.073***
	(0.063)	(0.063)	(0.066)	(0.015)
4. East residence	0.148***	0.169***	0.237***	0.144***
	(0.034)	(0.037)	(0.036)	(0.009)
5. Constant	2.031***	1.893***	2.035***	0.746***
	(0.085)	(0.107)	(0.336)	(0.099)
Observations	14,804	14,804	14,804	14,804
*R* ^2^	0.064	0.076	0.176	0.290
Distance	Yes	Yes	Yes	Yes
PEA traffic	Yes	Yes	Yes	Yes
Near angle FE	Yes	Yes	Yes	Yes
Draw controls	Yes	Yes	Yes	Yes
Block FE	No	Yes	Yes	Yes
Demography	No	Yes	Yes	Yes
Other exposures	No	Yes	Yes	Yes
SES	No	No	Yes	Yes
Timing controls	No	No	Yes	Yes
Person RE	No	No	Yes	Yes

Notes: Bootstrapped SE in parentheses *** *P* < 0.01, ** *P* < 0.05, and * *P*< 0.1; In columns (1) to (3) BLL is in μg/dL; ^†^, in column (4), we take the natural log of BLL. All models limited to children ≤5 y of age, residing <1.5 miles RHV (or 2.4 km); Distance is defined between RHV and the child’s residence; Residential near angle is defined in equation [1], with east residence being downwind children; PEA traffic is average daily PEA operations at RHV, calculated over 60 days from child’s date of draw and normalized. Demography includes child’s age (years) and sex (1=female, 0=otherwise); Draw controls includes: draw method (1=capillary, 0=otherwise), limit of quantification (1=BLL ≤ limit of quantification, 0=otherwise), and repeated sample (0=singleton observation, 1,...,*n*=repeated *n* times); Other exposures includes: count of TRI facilities ≤2 miles from residential address, and percent of neighborhood housing stock built ≤ 1960; SES is the neighborhood socioeconomic status index; timing controls include indicators for season and year-quarter of the date of draw; inclusion of variables is denoted yes, where applicable.

With respect to distance, reported coefficients in Table 1 have the interpretation of an estimated difference in mean BLLs (in μg/dL units) for children at 0.5 to 1 mile (or 0.8 km to 1.6 km) and 1 to 1.5 miles (or 1.6 km to 2.4 km), respectively, vis-a-vis children most proximate to northwest tip of RHV (point coordinates 37.336225, −121.8230194) (Supplementary Material Table S2 reports results involving the estimation of a series of linear models with residential distance measured continuously and applying various transformations to both distance and child BLLs. Other things held equal, we find that no matter the measurement or transformation—distance measured linearly, log or square root transformed and child BLLs measured linearly or log transformed—child BLLs decrease statistically significantly with residential distance from RHV).

For residential near angle, the east parameter estimate has the interpretation of an estimated difference in mean BLLs (in μg/dL units) for sampled children residing east (and predominantly downwind), relative to sampled children north of RHV. PEA traffic exposure is measured as a rolling average of PEA operations over 60 days from the date of a child’s blood draw. This quantity is converted to a percentile ranging from 0 to 1. With respect to PEA traffic, coefficients have the interpretation of the estimated change in child BLLs (in μg/dL units) associated with an increase in PEA traffic exposure from the observed minimum to the maximum.

We report coefficients from four different models that graduate in their saturation of control variables. The coefficients pertaining to our indicators of risk behave relatively consistently across models of varying saturation. Model (4) reports coefficients involving the natural log transformation of child BLL. Focusing our interpretation on models (3) including all possible control variables, we find that sampled children at 0.5 to 1 mile and 1 mile to 1.5 miles present with BLLs that are 0.234 and 0.235 μg/dL lower on average than sampled children nearest to RHV(< 0.5 miles). With respect to residential near angle, in model (3) we find that sampled children residing east (and predominately downwind) have BLLs that are 0.237 μg/dL higher than sampled children north of RHV. As shown in model (3), child BLLs are responsive to the measured volume of PEA traffic, increasing an estimated 0.168 μg/dL with an increase in PEA traffic exposure from the observed minimum to the maximum of traffic.

To contextualize the meaning of estimated differences in BLLs by distance, near angle, and traffic exposure, we compare our results to the estimated increase in BLLs of children in Flint during the much publicized Flint Water Crisis (FWC). At the height of the FWC, child BLLs surged by an estimated 0.35 to 0.45 μg/dL over baseline levels (15) (With over 21,000 time-stamped blood lead samples from children in Genesee County drawn from 2013 January 01 to 2016 July 19, (15) pursued a series of quasi-experimental tests to identify the causal effects of water-lead exposure, finding that the switch in water source in Flint caused child BLLs to increase by about 0.35 to 0.45μg/dL from a precrisis baseline of about 2.3 μg/dL). As shown in Table 1, children within 0.5 miles of RHV, children east of RHV, and children exposed to maximum traffic have BLLs that are about 0.2 μg/dL higher than statistically similar children more distant from RHV, residing north of RHV, and exposed to minimum traffic, respectively. These estimated differences are equivalent to about 50% of the estimated increase in BLLs of sampled children at the height of the FWC over baseline levels in Flint.

Next, we analyze threshold effects. Table 2 reports odds ratios for our main indicators of avgas exposure risk across three models with varying saturation of control variables. Given the ordered categorical measurement of our response variable, the reported odds ratios have the interpretation of the expected change in the odds of a child’s blood lead sample exceeding 4.5 μg/dL relative to the combined odds of appearing in lower BLL categories. Focusing on saturated model (3), as compared to children <0.5 miles of RHV, sampled children residing 0.5 to 1 mile from RHV have 0.827× lower odds of superseding 4.5 μg/dL relative to the combined odds of lower BLL categories. For children at 1 to 1.5 miles, the probability of a blood lead sample exceeding 4.5 μg/dL is 21.4% lower than statistically similar children at <0.5 miles. With respect to residential near angle, children residing east of RHV are 2.18× more likely to present with BLLs ≥4.5 μg/dL than children residing north of RHV, all else held equal. On the question of PEA traffic exposure, we find that an increase from minimum to maximum exposure increases the odds of eclipsing 4.5 μg/dL relative to the combined odds of presenting with a lower BLL category by a multiplicative factor of 1.31.

**Table 2.** Proportional odds of residential distance, near angle, and PEA Ttraffic vis-à-vis categorical child BLLs.

	(1)	(2)	(3)
1. Distance RHV (0.5 to 1 miles)	0.847**	0.828**	0.827**
	(0.060)	(0.070)	(0.072)
2. Distance RHV (1 to 1.5 miles)	0.819***	0.804***	0.786***
	(0.055)	(0.066)	(0.068)
3. PEA traffic volume	1.989***	2.045***	1.311***
	(0.111)	(0.118)	(0.099)
4. East residence	1.749***	1.828***	2.182***
	(0.119)	(0.147)	(0.218)
Observations	14,804	14,804	14,804
Distance	Yes	Yes	Yes
PEA traffic	Yes	Yes	Yes
Near angle FE	Yes	Yes	Yes
Draw controls	Yes	Yes	Yes
Block FE	No	Yes	Yes
Demography	No	Yes	Yes
Other exposures	No	Yes	Yes
SES	No	No	Yes
Timing controls	No	No	Yes
Person RE	No	No	Yes

See Table 1 Notes.

### Extended analysis

While results reported in Table 1 and Table 2 on child residential distance, residential near angle, and exposure to PEA traffic support the hypothesis that child BLLs are statistically associated with the risk of exposure to avgas, next we report results from additional analyses involving the statistical interaction of residential distance and PEA traffic, a natural experiment involving an observed contraction in PEA aircraft at RHV following social distancing measures enacted countywide, and the substitution of PEA traffic with measured atmospheric concentrations of lead at the airport.

First, we consider a statistical interaction between PEA traffic exposure and residential distance. Insofar as avgas gasoline exposure is a source of risk, we expect that the BLLs of sampled children proximate to RHV will be more responsive to the flow of PEA traffic than children more distant from the airport. As before, Table 3 presents coefficients for different models that increase successively in the saturation of control variables. Across models (1) through (4), estimated coefficients behave as theoretically expected and are distinguishable from chance. Model (4) reports coefficients involving the natural log transformation of child BLL. Concentrating interpretation on model (3), the main effect of residential distance indicates that sampled children at 0.5 to 1.5 miles (or 0.8 to 1.6 km) from RHV present with BLLs that are 0.242 μg/dL lower than children nearest to the airport. Because PEA traffic is centered at the mean, the coefficient on PEA traffic exposure indicates that a doubling of PEA traffic from the mean is associated with a 0.845 μg/dL increase in child BLLs, all else held equal. The estimated coefficient of interaction is negative ($\widehat{\delta }\, =$ -0.720), implying that an increase in PEA traffic exposure affects the BLLs of sampled children more distant from RHV less than children proximate to RHV.

**Table 3.** Coefficients of PEA traffic × residential distance at RHV vis-à-vis child BLLs.

BLLs (μg/dL)	(1)	(2)	(3)	(4)^†^
1. Distance RHV (0.5 to 1 miles)	−0.175***	−0.241***	−0.242***	−0.059***
	(0.066)	(0.079)	(0.064)	(0.018)
2. PEA traffic volume	1.080***	1.034***	0.845***	0.235***
	(0.219)	(0.211)	(0.182)	(0.051)
3. Distance RHV × PEA traffic	−0.817***	−0.760***	−0.720***	−0.173***
	(0.227)	(0.220)	(0.195)	(0.051)
4. Constant	2.196***	2.063***	2.139***	0.789***
	(0.083)	(0.109)	(0.325)	(0.096)
Observations	14,804	14,804	14,804	14,804
*R* ^2^	0.065	0.077	0.177	0.291
Distance	Yes	Yes	Yes	Yes
PEA traffic	Yes	Yes	Yes	Yes
Near angle FE	Yes	Yes	Yes	Yes
Draw controls	Yes	Yes	Yes	Yes
Block FE	No	Yes	Yes	Yes
Demography	No	Yes	Yes	Yes
Other exposures	No	Yes	Yes	Yes
SES	No	No	Yes	Yes
Timing controls	No	No	Yes	Yes
Person RE	No	No	Yes	Yes

See Table 1 Notes.

**Fig. 1.**
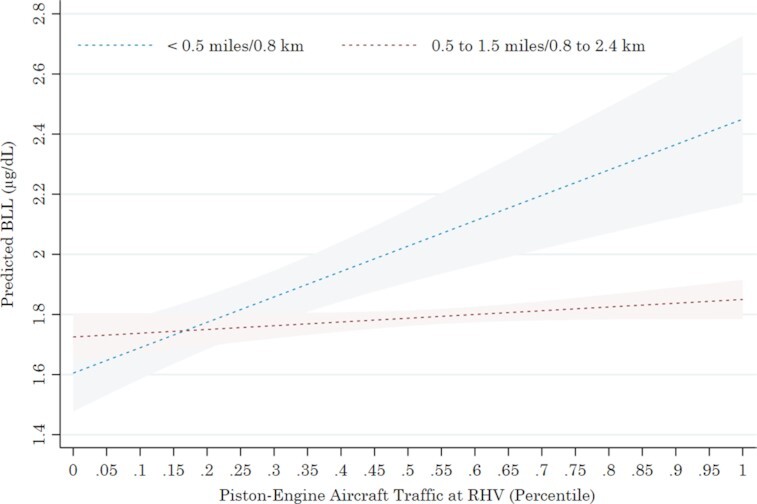
Predicted child BLLs by residential distance and PEA traffic.

Figure 1 visualizes the effects reported in Table 3, showing predicted BLLs of sampled children at two distances—within 0.5 miles (0.8 km) and 0.5 to 1.5 miles from RHV—over the range of observed PEA traffic exposure. Predictions are from model (3) in Table 3, with all other model covariates set to their means. Figure 1 shows that, all else held equal, a movement from the minimum to the maximum PEA traffic exposure increases the BLLs of sampled children proximate to RHV by 0.92 μg/dL (1.57 to 2.49 μg/dL). By comparison, children more distant from RHV (0.5 to 1.5 miles) experience a more modest increase in BLLs of about 0.16 μg/dL (1.71 to 1.87 μg/dL) for an increase in PEA traffic from the minimum to the maximum.

The interaction effect of piston engine aircraft traffic exposure and residential distance persists when we restrict the sample to toddlers (age 12 to 24 months), that are especially vulnerable to place-based exposures (16). Recapitulating the results of model (3) in Table 3 and limiting to sampled children age 12 to 24 months, we observe an amplification of the distance × traffic effect. The BLLs of sampled toddlers living near RHV increase by 1.60 μg/dL (1.79 to 3.39 μg/dL) with a change from minimum to maximum exposure to PEA traffic (see Supplementry Material Figure S1). Sensitivity tests in which PEA traffic is substituted for monthly quantities of avgas sold at RHV, behave similarly. In going from 5,000 to 35,000 gallons of avgas sold, the BLLs of children who live near the airport increase by an estimated 0.54 μg/dL (see Supplementary Material Figure S2).

Next, we present results of a robustness test that leverages reductions in aircraft traffic following the outbreak of COVID-19. As the pandemic gripped the country, state and local governments enacted various restrictions on the behavior of households and firms to limit the spread of the disease. Corresponding with these efforts, PEA traffic declined measurably at RHV over the months of February to July of 2020. Compared to three baseline control periods—2011 to 2019, 2015 to 2019, and 2018 to 2019—PEA traffic declined by 34% to 44%. PEA traffic at RHV returned to pre-pandemic levels in August to December of 2020. The pandemic-caused dynamics in PEA operations at RHV present us with a natural experiment. If avgas exposure is a source of risk, then we should observe a reduction in the BLLs of children sampled in this PEA traffic contraction period, other things held equal. Table 4 presents estimated coefficients pertaining to the PEA traffic contraction period. As expected, the BLLs of sampled children during the PEA traffic contraction are significantly lower vis-à-vis children sampled before and after the contraction. Across models (1) and (2), we find that BLLs decreased by about 0.23 μg/dL, depending on the presence of control variables. The coefficient attenuates intuitively with the inclusion of measured PEA traffic exposure in model (3) and in model (4) where child BLLs are log transformed.

**Table 4.** Coefficients of PEA traffic contraction period at Reid-Hillview vis-à-vis Child BLLs.

BLLs (μg/dL)	(1)	(2)	(3)	(4)^†^
1. Contraction period	−0.236***	−0.230***	−0.102*	−0.037**
	(0.032)	(0.034)	(0.061)	(0.071)
2. Constant	2.187***	2.082***	1.964***	0.721***
	(0.084)	(0.107)	(0.362)	(0.100)
Observations	14,804	14,804	14,804	14,804
*R* ^2^	0.062	0.074	0.176	0.290
Distance	Yes	Yes	Yes	Yes
PEA traffic	No	No	Yes	Yes
Near angle FE	Yes	Yes	Yes	Yes
Demography	Yes	Yes	Yes	Yes
Draw controls	Yes	Yes	Yes	Yes
Block FE	Yes	Yes	Yes	Yes
Other exposures	No	Yes	Yes	Yes
SES	No	No	Yes	Yes
Timing controls	No	No	Yes	Yes
Person RE	No	No	Yes	Yes

See Table 1 Notes.

Last, we evaluate the relationship between child BLLs and measured atmospheric concentrations of lead at the airport with data from the Bay Area Air Quality Management District (BAAQMD). The BAAQMD data covered the period of 2012 February to 2018 March, with an atmospheric reading taken (on average) every 6 days. The monitor was located in the aircraft run-up zone (point coordinates, 37.329841, −121.815438). Given the time-abbreviated nature of the air quality data, only 9,542 of the 14,804 blood lead samples used in our analysis could be assigned an atmospheric lead concentration coincident with the timing of blood draw. Results are reported in Table 5. Focusing attention on Model (2), an increase in atmospheric lead of 1 microgram per cubic meter (μg/m^3^) increases child BLLs by 4.05 μg/dL (As noted in the methods section, this observed effect corresponds to a measurement of atmospheric lead involving a twomonth moving average (in micrograms per cubic meter) from the date of child blood draw. Restricting to 30 days before blood drawreduces the estimated coefficient to 2.45 μg/dL (95% CI:0.93, 3.96)). More substantively, an increase from the observed minimum to the observed maximum (of 0.04 to 0.12 μg/m^3^) is associated with an increase of about 0.21 μg/dL, an effect size comparable to what we observe with respect to measured PEA traffic. Intuitively, in model (4), the observed atmospheric concentration effect dissipates with the inclusion of measured traffic. Following Richmond-Bryant et al (17), we also render a version Eq. (8) that takes the natural log of child BLL and atmospheric lead. Our estimated elasticity of child BLL vis-a-vis atmospheric lead of 0.123 (95% CI:0.075, 0.170) matches Richmond-Bryant et al (17) near exactly (see Supplementary Material Figure S3).

**Table 5.** Coefficients of atmospheric lead concentrations vis-à-vis child BLLs.

BLLs (μg/dL)	(1)	(2)	(3)^†^	(4)
1. Atmospheric lead (μg/m^3^)	4.312***	4.054***	1.625***	2.102
	(1.289)	(1.300)	(0.348)	(1.372)
2. Constant	1.470***	1.238**	0.086	−0.676
	(0.403)	(0.470)	(0.164)	(0.630)
Observations	9,542	9,542	9,542	9,542
*R* ^2^	0.262	0.266	0.266	0.268
Distance	No	Yes	Yes	Yes
Near angle FE	No	Yes	Yes	Yes
PEA traffic	No	No	No	Yes
Draw controls	Yes	Yes	Yes	Yes
Block FE	Yes	Yes	Yes	Yes
Demography	Yes	Yes	Yes	Yes
Other exposures	Yes	Yes	Yes	Yes
SES	Yes	Yes	Yes	Yes
Timing controls	Yes	Yes	Yes	Yes
Person RE	Yes	Yes	Yes	Yes

Dependent variable is BLL in μg/dL; See Table 1 Notes.

## Discussion

In this study, we assessed whether the BLLs of sampled children around RHV are associated with indicators of aviation-related lead exposure, net of other lead exposure pathways.

### Main analysis

Controlling for other known sources of lead exposure both explicitly and indirectly (As described in the methods section on control data, statistical models adjust for child proximity to lead-emitting toxic release inventory facilities, legacy use of lead-based paint by measurement of the age of housing stock in the census tract of residence, and include a neighborhood fixed effect to account for unobservables like soil lead accumulation that may influence BLLs that are common to sampled children within a given neighborhood but varying across neighborhoods), demographic characteristics, and neighborhood conditions, the evidence from main analyses of a statistical link between avgas exposure risk and child BLLs includes:

1. The BLLs of the sampled children increase significantly with proximity to RHV. Children residing within 0.5 miles (0.8 km) of RHV present with significantly higher BLLs than children more distant of RHV. As shown Supplementary Material Table S2, this relationship between child BLLs and distance to RHV Airport is robust to various linear and nonlinear transformations of both input and response variables.
2. BLLs are significantly and substantively higher among sampled children residing East (and predominantly downwind) of RHV.
3. BLLs of sampled children increase significantly with the volume of measured PEA traffic at RHV from the date of blood draw.
4. As evidenced in Table 2 the probability that a sampled child’s BLL exceeds the CDPH-defined threshold of 4.5 μg/dL, increases significantly with proximity to RHV, is higher among children residing east of RHV, and increases with the volume of PEA traffic.

Estimated relationships between BLLs and our main indicators of avgas exposure risk are quantitatively similar to results of other studies (10,11).

### Extended analysis

Again, controlling for other known sources of lead, child demographic characteristics, and neighborhood conditions, the evidence for a statistical link between child BLLs and avgas exposure from extended analyses, include:

1. As evidenced in Table 3, the BLLs of sampled children proximate to RHV are significantly more responsive to PEA traffic and avgas sales at RHV (see Supplementary Material Figure S1) than quantitatively similar children who live more distant from the airport. Substantively, an increase from minimum to maximum PEA traffic increases the BLLs of proximate children by over 0.70 μg/dL.
2. The interaction effect of child residential distance and volume of PEA traffic amplifies for toddlers 12 to 24 months, a particularly sensitive subpopulation to place-based exposure risk.
3. Following efforts to stem the spread of COVID-19, PEA traffic declined significantly in the months of February to July at RHV. As evidenced in Table 4, the BLLs of children sampled in this PEA traffic contraction period declined significantly.
4. As shown in Table 5, statistically significant results persist with the substitution of PEA for measured atmospheric concentration of lead at the airport. Our estimated elasticity of child BLL vis-a-vis atmospheric lead corroborates Richmond-Bryant et al (17) finding that child BLLs increase with exposure to airborne lead concentrations (TSP) below 0.15 μg/m^3^.

While it is statistically improbable that the ensemble of evidence presented above arises by chance alone, we briefly consider a possible objection arising from child residential proximity to the San Jose Speedway (SJS). The SJS operated for many decades and was located southwest of RHV (see Supplementary Material Figure 4) (We wish to thankMichael McDonald for alerting us to the history of the SJS and for forwarding this hypothesis). Importantly, the cars racing the oval at SJS were fueled with lead-formulated gasoline. In a clever natural experiment exploiting the switch from leaded to unleaded gasoline in NASCAR and ARCA racing series in 2007, Hollingsworth and Rudik (2019) (18) found that “(i) ambient airborne lead concentrations increase immediately after a NASCAR race, (ii) counties with leaded NASCAR races have higher rates of child lead poisoning.” Additionally, Bui et al (2022) (19) found that maternal exposure to airborne lead emissions from NASCAR races produced significant adverse pregnancy outcomes. Perhaps, these acute NASCAR effects have a lasting legacy, with the lead emitted from racing events depositing in the soils of neighborhoods of where children now reside. To test this possibility, we calculated the Haversine distance from a sampled child’s residence to the historic location of the SJS (point coordinates 37.3293856, −121.8202305), see Supplementary Material Figure S4 for aerial photo. As we do for distance to RHV, we test the effect of distance to the SJS in both continuous and categorical terms of <0.5 miles, 0.5 to 1 mile, 1 to 1.5 miles, and >1.5 miles (see Supplementary Material Table S3).

Supplementary Material Table S3 shows results from this exercise, with distance to the speedway measured continuously and categorically, and with and without indicators of avgas exposure risk emanating from RHV. Across all models, the effect of proximity to the historic SJS on child BLLs is indistinguishable from chance. Because the historic location of the SJS is west of RHV, the null results are compatible with our finding showing that the BLLs of sampled children west (and predominately upwind) of RHV have lower BLLs than children east (and predominately downwind) of RHV.

As noted in the methods section, our point location decision at the northwest end (The northwest corner of RHV is also home to aircraft maintenance activities known to release lead in significant enough quantities to increase the risk of elevated blood lead in workers and indirectly among children in their care. Chen and Eisenberg (2013) (20) report that “The airborne lead concentration during sandblasting of spark plugs approached an occupational exposure limit for a short-term exposure, [with] small parts, tools, and metal shavings on and around workbench areas, desktops, and open shelving units pos[ing] a safety hazard.”) of the airport was motivated by previous research showing that the bulk of emissions released over the landing-takeoff (LTO) cycle occur at take-off and climb out (8). Pointing to a recently published EPA report with model-extrapolated estimates of airborne lead at RHV, readers may note Section C.2.2 and accompanying figures C-3 to C-5 showing that ground-level lead concentrations appear to collect disproportionately at the Southeast corner of RHV during the run-up phase of the LTO cycle. While very important to the study of ground-level emissions, Carr et al (2011), Feinberg and Turner (2013) (21), and the EPA report itself (2020) (14) note that run-up emissions only account for about 11% of all airport lead emissions.

Still, to address possible concerns that our findings result from our point location decision, we perform a series of analyses involving various other point locations at the airport. Each new point location analyzed required separate distance and near angle calculations to a sampled child’s place of residence. Supplementary Material Table S4 summarizes this statistical exercise. Across all models, the coefficients pertaining to child residential distance, near angle, and PEA traffic are robust to the point location judgment.

On the matter of avgas exposure risk to families and children proximate to general aviation airports, the National Academies of Sciences, Engineering, and Medicine maintains: “Because lead does not appear to exhibit a minimum concentration in blood below which there are no health effects, there is a compelling reason to reduce or eliminate aviation lead emissions.” The ensemble evidence compiled in this study supports the “compelling” need to limit aviation lead emissions to safeguard the welfare and life chances of at-risk children.

## Materials and methods

### Child blood lead data

Permission to analyze blood lead was granted by agreement with the Childhood Lead Poisoning Prevention Branch (CLPPB) of the California Department of Public Health (CDPH). Databases were queried for records with: (1) an indication of residence in Santa Clara County, (2) a date of blood draw occurring within the last 10 y, (3) a date of birth for the sampled person, and (4) a reported blood lead value.

CDPH-records with indication of a residential address in Santa Clara County were independently geo-coded. We normalized each residential address by removing special characters and apartment numbers or letters. The resulting query parameter of this process was a lowercase string in the form of “number street, city, state” that was submitted to the Google Geocode API service to derive longitude and latitude point coordinates for each address record.

Responses from the API service included a confidence label indicating the level of accuracy, with the highest level of accuracy being a “rooftop” match. In all, 94.28% of address records were uniquely matched to rooftop point coordinates. Unmatched addresses were excluded from the final data set. Point coordinates corresponding to each rooftop address was then used to calculate distance and near angle variables. Restricting to children ≤5 y of age at the time of blood draw, residing < 1.5 miles (or 2.4 km) of RHV, observed from 2011 January 1 to 2020 December 31, and with a rooftop address, we arrived at 14,876 blood lead sample observations for this statistical analysis.

The main response or outcome variable of analytic interest is BLL) measured in micro-grams per deciliter of blood (μg/dL units). Restricting to children ≤5 y of age at the moment of blood sample, residing <1.5 miles of Reid-Hillview, and observed from 2011 January 1 to 2020 December 31, the unconditional mean BLL of sampled children was 1.80 μg/dL. About 1.5% of sampled children present with BLLs ≥ 4.5μg/dL, the CLPPB-defined threshold for action.

Five control variables from RASSCLE II/HL7 known to be correlated with child BLLs were collected from CDPH data, including: child gender, child age, method of blood draw, sample detection limit, and sample order. Gender is measured as $1\, =$ female; child *age* is measured in years (ranging from 0 to 18); the method*of*blood draw $=\, 1$ if capillary, and $0 \, = \,$ otherwise; sample detection limit is measured as $1\, =$ if the reported BLL is at or below the limit of quantification, and $0 \, = \,$ otherwise (In Supplementary Material Table S5 we render a series models where the observed BLLs is adjusted by common single imputation methods involving 1) BLL/ √ 2; 2) BLL×log 2; and ln(BLL/ √ 2)); and sample order which codes the count of blood samples (0=singleton observation, 1,...,*n* = repeated *n* times).

### Avgas exposure risk data

We test three independent indicators of exposure risk to leaded avgas, including child residential distance, child residential near angle to capture whether a sampled child resides downwind of RHV, and the volume of PEA traffic from the moment of child blood draw. Child exposure risk to leaded avgas (and implied dispersion of the pollutant) is assumed to decrease linearly with distance, increases with downwind residence, and increases linearly with measured volume of PEA traffic.

#### Residential distance

Following others (10,11), we calculate the distance from the residential address of a sampled child to RHV. Using distance information on each child as an indicator of exposure risk, we test whether the BLLs of sampled children increase measurably with proximity to RHV.

Over the LTO cycle, studies find that the bulk of aircraft emissions are released during departure phases of run-up, takeoff, and climb-out (22–24). According to (8), total fuel consumed by piston aircraft in departure phases of the LTO cycle is estimated at 82% for twin-engine aircraft and 85% for single-engine aircraft. About 80% of lead emissions are released during departure phases of the LTO cycle (8).

Given that the bulk of lead emissions are released during departure phases of the LTO cycle, we capture child proximity by calculating the Haversine distance (The Haversine of the central angle, which is d over the r, is calculated by: $\left(\frac{d}{r}\right)$ = haversine( 2 − 1) + cos( 1)cos( 2)haversine(λ2 − λ1), where r is the radius of earth(6,371 km), d is the distance between a child’s residence and RHV, φ1, φ2 is latitude and λ1, λ2 is longitude of the child’s residence and Reid-Hillview, respectively. We solve for d by the inverse sine function, getting: d = rhav−1(h) = 2rsin−1( √ h)) from the child’s residence at the date of blood draw to the northwest tip of RHV (point coordinates 37.3362252, -121.8230194). In addition to measuring distance continuously, residential distance is also divided into three even categories: < 0.5 miles (0.8 km), 0.5 to 1 mile (0.8 to 1.6 km), and 1 to 1.5 miles (1.6 to 2.4 km) from RHV.

Over the period of 2011 January 1 to 2020 December 31, we observe a total of 930 records at <0.5 miles, 5,564 records at 0.5 to 1 mile, and 8,382 at 1 to 1.5 miles from RHV. Insofar as avgas exposure is a source of risk, sampled children in the nearest orbit to RHV should present with higher BLLs as compared to sampled children in outer orbits. Sampled children in our inner orbit of <0.5 miles of are statistically similar to children in outer orbits (0.5 to 1.5 miles) with respect gender, residential near angle, age, PEA traffic exposure, sample order, year or timing of blood draw, and proportion of children sampled by capillary method where *P* > 0.05. We do observe statistically significant differences with respect to the percentage of neighborhood homes built prior to 1960 (24.1 vs 28.2, *P* < 0.001), the count of lead-emitting toxic release inventory facilities within 2 miles of a child’s residence (2.37 vs 2.51, *P* < 0.001), and neighborhood socioeconomic status (−0.22 vs −0.27, $\mathit{ P}\, =$ 0.007). On variables where statistically significant differences are observed, all function to inflate the BLLs of sampled children in outer orbits. Therefore, whatever differences in estimated BLLs that may obtain between sampled children by residential distance in regression analyses we may regard these differences as possibly attenuated.

#### Residential near angle

The fate and transport of lead emissions depend on the direction of prevailing winds that vary in and across airport facilities. Insofar as avgas is an independent source of lead exposure, two children equidistant to the same airport face different risk of elevated blood lead depending on the child’s residential near angle to the airport.

A near angle group was assigned to each address by calculating the compass bearing (degrees) between a child’s residential location and RHV. We define near angle groups by the four cardinal directions: North (N), East (E), South (S), and West (W). For a BLL sample from child *i* in time *t*, with range of possible compass bearings *b*_*it*_ ∈ [0, 360), we assign near angle group *a*_*it*_ as:

(1) \begin{eqnarray*} a_{it} = \ \left\lbrace \begin{array}{@{}l@{\quad }l@{}}E, \quad &\text{if } b_{it} \in \left[ 45^{\circ }, 135^{\circ } \right), \\ S, \quad &\text{if } b_{it} \in \left[ 135^{\circ }, 225^{\circ } \right), \\ W, \quad &\text{if } b_{it} \in \left[ 225^{\circ }, 315^{\circ } \right), \\ N, \quad &\text{otherwise.} \end{array}\right. \end{eqnarray*}

Because the direction of prevailing winds at RHV emanate from the West and Northwest, and insofar as exposure to avgas is a source of risk, children residing east of the airport ought to present with higher BLLs (see Supplementary Material Figure S5 for distribution of sampled children by near angle grouping).

#### PEA traffic and avgas sales

The volume of PEA traffic varies meaningfully between airports and within an airport in time. Therefore, two children residing in the same household but sampled at different moments in a calendar year may present with different BLLs, depending on the coincidence of PEA traffic. To capture this channel of risk, we collected data on PEA departures and arrivals from TMSC.

Daily PEA data were available for RHV. Because the half-life for lead in blood is about 30 days (25), we back-calculated a rolling average of PEA operations over 60 days from the date of a child’s blood draw. In Supplementary Material Table S6 we present results with our measure PEA traffic divided into terciles, showing an apparent dose-responsivity of child BLLs vis-a-vis PEA traffic. With the date of blood draw linked to the quantity of PEA traffic, one can test whether child BLLs are dose-responsive with the volume of PEA traffic. Our measurement of PEA traffic exposure assumes that children have continuity of residence for 60 days.

Also, fuel flowage fee (FFE) data were obtained from personnel at the Roads and Airports Department of Santa Clara County. The FFE data track monthly quantities of avgas (100LL) sold to fixed-base operators at RHV from 2011 to 2019. Each child is matched to the 2-month rolling average of quantities of 100LL sold from the date of blood draw. As with PEA traffic, we test whether child BLLs are dose-responsive with avgas sales at RHV.

#### Control data

Lead-emitting industrial facilities are more common in the vicinity of airports (11).

Children that are proximate to airports are therefore simultaneously proximate to other point-source emitters of lead. Failing to account for this spatial coincidence can produce biased estimates of avgas exposure risk vis-à-vis BLLs in children. The U.S. EPA’s TRI system tracks the industrial management of over 650 listed chemicals that pose harm to humans and the environment. We collected records on all facilities in Santa Clara County with reported on-site releases of lead between 2011 and 2020. Following (11), with the location of each facility and the year of reported release event, we counted the number of lead-emitting TRI facilities ≤2 miles (or 3.2 km) of a child’s residence in the corresponding year of blood draw. All results pertaining to the assessment of statistical relationships of child BLLs and indicators of avgas exposure risk control for the presence of this alternative source of lead exposure.

Legacy use of lead-based paint remains an exposure risk to children. Exposure to lead-based paint is primarily a problem in older homes. By 1960, use of lead-based paint subsided by more than 90% from peak usage in the 1920s. Still, children in the United States may ingest paint chips or may be exposed to dust from deteriorating or haphazardly removed lead-based paint in homes built in the era before 1960. We collected American Community Survey data on the fraction of homes in a child’s neighborhood built before 1960. In the analyses that follow, each sampled child in our data is assigned a lead-based paint exposure risk according to the neighborhood of residence and year of blood draw, as captured by the percentage of homes built before 1960.

Studies show that children of low socioeconomic status are at greater risk of presenting with elevated BLLs (26,27). Socioeconomic status proxies for household resources, knowledge about the dangers of, and protective actions taken against, lead exposure (11). In addition to demographic information present in CDPH data, we measured the percentage of adults with a college degree, median home prices, and median household incomes to characterize the socioeconomic status of a child’s residential neighborhood. These data were also collected from the American Community Survey. Supplementary Material Table S1 provides descriptive statistics.

### Empirical methods

To assess whether the BLLs of sampled children are statistically associated with indicators of avgas exposure risk, we deploy a linear least squares estimator with census block fixed effects, accounting for heteroeskedasticity and relaxing distributional assumptions with bootstrapped SE.

The outcome of interest is child BLL, measured as a continuous variable in μg/dL (and the natural log of child BLL). For sampled child *i* in neighborhood block *j* at time *t*, we estimate the responsiveness of child blood lead *Y*_*ijt*_ to indicators of avgas exposure risk with the following linear model

(2) \begin{eqnarray*} \mathit{ Y}_{ijt} &=& \beta _{0} + \beta _{1} D_{it}^{n} + \beta _{2} D_{it}^{f} + \beta _{3} T_{it} + \beta _{4} W_{it}^{e} + \beta _{5} W_{it}^{s} + \beta _{6} W_{it}^{w}\\ && + \Gamma _{1} G_{i} + \Gamma _{2} A_{it} + \Gamma _{3} C_{it} + \Gamma _{4} S_{i} + \Gamma _{5} Z_{it} + \Gamma _{6} L_{it}\\ && + \lambda _{1} F_{it} + \lambda _{2} H_{jt} + \lambda _{3} I_{jt} + \lambda _{4} Q_{it} + \gamma _{i} + \gamma _{j} + \varepsilon _{ijt} . \end{eqnarray*}

Knowing that relationships of interest are possibly nonlinear, we use a flexible specification where distance *D* is measured as a series of dichotomous variables, where $D_{it}^{n} \, = \, 1$ if child *i* in time *t* resides 0.5 to 1 miles from RHV, $0 \, =$ otherwise, and $D_{it}^{f} \, = \, 1$ if child *i* in time *t* resides 1 to1.5 miles from RHV, and 0 otherwise. Children most proximate to RHV (<0.5 miles) constitute the reference distance. The flow of lead emitted from the aircraft traffic *T*_*it*_ is the count of PEA operations (measured in percentile terms) in the last 60 days relative to the draw date *t* of child *i*. To account for prevailing wind direction we include a series of dummy variables *W* for the location of child *i* in time *t* relative to the airport, with North being the reference direction, and: $W_{it}^{e} \, = \, 1$ if a child resides East of RHV, $0 \, = \,$ otherwise, $W_{it}^{s} \, = \, 1$ if a child resides South of RHV, $0 \, = \,$ otherwise, and $W_{it}^{w} \, = \, 1$ if a child resides West of RHV, $0 \, = \,$ otherwise.

A series of variables are included to control for the timing, method, quantification limit, and order of blood draw, where *C*_*it*_ is whether or not the method of blood draw is capillary, *L*_*it*_ is whether the measured BLL is at or below the limit of test detection, *Z*_*it*_ is the year and quarter of the blood draw, and *S*_*i*_ is the order of sample for children sampled repeatedly (For a singleton observation (nonrepeated child) i, Si = 0. Otherwise, Si = 1, ..., n for child i repeated n times over the observation period, 2011 January 1 to 2020 December 31. The date of birth, child sex, child name, and date of blood draw were used to identify sample order for each child. The majority of children (53.3%) appearing in CDPH data were sampled only once). Child demographic characteristics include the child’s age *A*_*it*_ measured in years, and an indicator for whether the child is female *G*_*i*_.

We include a suite of controls to account for confounding sources of lead exposure and neighborhood socioeconomic status corresponding to the residential location of a sampled child and the date of blood draw. *F*_*it*_ is the count of nearby lead-emitting toxic release inventory facilities ≤ 2 miles of a child’s residence, and *H*_*jt*_ is the percent of homes built ≤ 1960 in child’s neighborhood of residence, proxying for lead-based paint exposure risk. Because atmospheric concentrations of lead fluctuate seasonally—in part because of the re-suspension of lead-contaminated surface soils by turbulence (28,29)—our statistical models proxy for this phenomenon with a series of dummy variables corresponding to the season of blood draw, *Q*_*it*_, with winter as our reference season. Also included is *I*_*jt*_, estimating the socioeconomic status of a neighborhood by an quantitative index that incorporates measures of educational attainment, median household income, and property values (proxying for household wealth).

Importantly, γ_*i*_ is the child random effect measured as the difference between the observed BLL and the child-specific average BLL and γ_*j*_ is the neighborhood or census block fixed effect. Inclusion of γ_*j*_ accounts for nontime varying unobservable factors, which may influence BLLs that are common to sampled children within a given neighborhood but varying across neighborhoods. Fixed effects absorb omitted variables by estimating a distinct mean BLL value (or intercept) for each neighborhood. Finally, *ε*_*ijt*_ is the random error term associated to the observed *Y*_*ijt*_.

#### Blood lead thresholds

We also reconstitute our response variable in ordered categorical terms, defining mutually exclusive BLL categories ranging from 0 to the exceedance of the CDPH-defined threshold of 4.5 μg/dL (For comparison, the current blood lead reference level set by the Centers for Disease Control and Prevention (CDC), adopted on 2021 May 14 is 3. μg/dL). The purpose here is to investigate threshold effects with respect to our main operations of avgas exposure risk and to relax the assumption of precisely measured BLLs, given uncertain laboratory test precision.

Under the premise that a given blood lead concentration is an imperfectly observed variable, we execute an ordered logistic regression, modeling BLL as a set of ordinal categories. Moving in increments of 1.5 μg/dL, we convert the continuous measure of blood lead concentration *Y*_*it*_ to a categorical variable *B*_*it*_, with cutpoints defined as

\begin{eqnarray*} B_{it} = \ \left\lbrace \begin{array}{@{}l@{\quad }l@{}}1, \quad &\text{if } Y_{it} \: \lt \: 1.5, \\ 2, \quad &\text{if } 1.5 \: \le \: Y_{it} \: \lt \: 3, \\ 3, \quad &\text{if } 3 \: \le \: Y_{it} \: \lt \: 4.5, \\ 4, \quad &\text{if } Y_{it} \: \ge \: 4.5, \end{array}\right. \end{eqnarray*}

where *Y*_*it*_ is in units of μg/dL (For sampled children within 1.5 miles of Reid-Hillview, we observe 6,489 records at<1.5μg/dL, 6,806 records at 1.5 to<3μg/dL, 1,361 records at 3 to <4.5 μg/dL, and 220 records at ≥ 4.5 μg/d).Within this framework, one can estimate the proportional odds a given blood lead concentration is in exceedance of a specified blood lead category. For child *i* with corresponding BLL observation in time *t*, *B*_*it*_ takes on the ordinal values *k* = 1,..., 4, then we define the cumulative response probabilities as

(3) \begin{eqnarray*} b_{itk} = \text{Prob}(B_{it} \le k | \mathbf {X}_{it}), \ \qquad k = 1,...,4 , \end{eqnarray*}

where $\mathbf {X}_{it}$ is a vector of explanatory values related to child *i* in time *t*. Using Eq. (3), we can represent a generalized logistic model as

(4) \begin{eqnarray*} {\rm logit}\; (b_{itk}) &=& {\rm ln}\; ( \frac{b_{itk}}{1 - b_{itk}} )\\ && = \theta _{k} + \mathbf {X}_{it}^{^{\prime }}\mathbf {\beta } , \end{eqnarray*}

where $\theta _{1} \le \theta _{2} \, ... \le \theta _{k}$. Taking the generalized model in Eq. (4) and the suite of covariates defined in Eq. (2), the fully specified model used to estimate the log-odds of sampled child *i* in neighborhood block *j* at time *t* being in BLL category *B*_*it*_ becomes

(5) \begin{eqnarray*} {\rm logit}\;( b_{ijtk}) &=& \theta _{k} + \beta _{1} D_{it}^{n} + \beta _{2} D_{it}^{f} + \beta _{3} T_{it} + \beta _{4} W_{it}^{e} + \beta _{5} W_{it}^{s}\\ && + \beta _{6} W_{it}^{w} + \Gamma _{1} G_{i} + \Gamma _{2} A_{it} + \Gamma _{3} C_{it} + \Gamma _{4} S_{i} + \Gamma _{5} Z_{it} + \Gamma _{6} L_{it}\\ && + \lambda _{1} F_{it} + \lambda _{2} H_{jt} + \lambda _{3} I_{jt} + \lambda _{4} Q_{it} + \gamma _{i} + \gamma _{j}, \qquad k = 1,...,4,\\ \end{eqnarray*}

Our expectation is that the exponentiated log-odds corresponding to $D_{it}^{n}$ and $D_{it}^{f}$ will be <1.0 reflecting lower risk of exceeding the threshold of 4.5 μg/dL among children in outer orbits of RHV relative to children nearest to RHV. We also expect that exponentiated log-odds corresponding $W_{it}^{e}$ to be >1.0, reflecting higher odds of maximum categorical blood lead for sampled children East of RHV relative to children North of RHV. Similarly, we expect the exponentiated coefficient on *T*_*it*_ to be >1.0, indicating that the risk of exceeding the CDPH-defined threshold of 4.5 μg/dL increases with exposure to PEA traffic.

#### PEA traffic exposure × residential distance

Next, we consider a statistical interaction between PEA traffic exposure and residential distance. Insofar as avgas exposure is a source of risk, we expect that the BLLs of sampled children proximate to RHV will be more responsive to the flow of PEA traffic than children more distant from the airport. Toward this analytic aim, we estimate the following

(6) \begin{eqnarray*} Y_{ijt} &=& \beta _{0} + \beta _{1} D_{it}^{nf} + \beta _{2} CT_{it} + \beta _{3} W_{it}^{e} + \beta _{4} W_{it}^{s} + \beta _{5} W_{it}^{w}\\ && + \delta \left( D_{it}^{nf} \times CT_{it} \right) + \Gamma _{1} G_{i} + \Gamma _{2} A_{it} + \Gamma _{3} C_{it} + \Gamma _{4} S_{i} + \Gamma _{5} Z_{it}\\ && + \Gamma _{6} L_{it} + \lambda _{1} F_{it} + \lambda _{2} H_{jt} + \lambda _{3} I_{jt} + \lambda _{4} Q_{it} + \gamma _{i} + \gamma _{j} + \varepsilon _{ijt}, \end{eqnarray*}

where, the meaning of all terms carry from Eq. (2) with the exception of $D_{it}^{nf}$ that now assumes a value of 1 if a sampled child resides in the outer orbit of 0.5 to 1.5 miles of RHV and 0 if a sampled child resides within 0.5 miles of RHV. Outer orbits are collapsed given insignificance of difference observed in Table 1. We expect *β*_1_ corresponding $D_{it}^{nf}$ to be negative, reflecting lower BLLs among distant children (0.5 to 1.5 miles) relative to proximate children (<0.5 miles). *CT*_*it*_ is the statistically centered value of PEA traffic exposure that is equal to $T_{it}-\bar{T_{it}}$ or the observed PEA traffic exposure (*T*_*it*_) minus the mean of PEA traffic exposure($\bar{T_{it}}$). We expect the corresponding parameter β_2_ to be positive, indicating that BLLs increase with the PEA traffic exposure. Finally, we expect δ corresponding to $D_{it}^{nf} \times CT_{it}$ to be negative, indicating that the BLLs of sampled children proximate to RHV (<0.5 miles) are more responsive to PEA traffic than children distant from RHV (0.5 to 1.5 miles).

#### PEA traffic contraction

As the COVID-19 pandemic gripped the country, state and local governments enacted various restrictions on the behavior of households and firms to limit the spread of the disease. Corresponding with these efforts, PEA traffic declined measurably at RHV over the months of February to July of 2020. As compared to three baseline control periods—2011 to 2019, 2015 to 2019, and 2018 to 2019 —PEA traffic declined by 34 to 44%. PEA traffic at RHV returned to pre-pandemic levels in August to December of 2020. The pandemic-caused dynamics in PEA operations at RHV present us with a natural experiment.

If as avgas exposure is a source of risk, then we should observe a reduction in the BLLs of children sampled in this PEA traffic contraction period, other things held equal. To test whether child blood levels behaved differently in the contraction moment, we estimate the following linear model

(7) \begin{eqnarray*} Y_{ijt} &=& \beta _{0} + \beta _{1} D_{it}^{n} + \beta _{2} D_{it}^{f} + \beta _{3} T_{it} + \beta _{4} W_{it}^{e} + \beta _{5} W_{it}^{s} + \beta _{6} W_{it}^{w}\\ && + \beta _{7} COV_{t} + \Gamma _{1} G_{i} + \Gamma _{2} A_{it} + \Gamma _{3} C_{it} + \Gamma _{4} S_{i} + \Gamma _{5} Z_{it} + \Gamma _{6} L_{it}\\ && + \lambda _{1} F_{it} + \lambda _{2} H_{jt} + \lambda _{3} I_{jt} + \lambda _{4} Q_{it} + \gamma _{i} + \gamma _{j} + \varepsilon _{ijt}, \end{eqnarray*}

where, all terms carry from Eq. (2) with the exception *COV*_*t*_ that is an indicator variable equal to 1 if a child is sampled in the PEA traffic contraction moment and 0 otherwise. Other things held equal, we expect the coefficient *β*_7_, corresponding to *COV*_*t*_, to be negative, indicating that children sampled in the PEA traffic contraction moment present with lower BLLs than children not sampled in this period (A reasonable concern with this analytic exercise is that the kind of children sampled in the PEA contractionmomentmay be characteristically different than children sampled outside this moment. Comparing means onmodel variables by children sampled in versus out of the PEA traffic contraction period, we find that sampled children are statistically indistinguishable in terms of residential distance to RHV (1.02 vs 1.03 miles, P = 0.515), fraction living east of RHV (0.07 vs 0.08, P = 0.178), child age (2.19 vs 2.09, P = 0.10), the proportion children that are female (0.49 vs 0.50, P = 0.691), and sample order (0.80 vs 0.82, P = 0.702). We do observe significant differences on the proportion of samples drawn by capillary method (0.25 vs 0.19, P < 0.001), the percentage of housing stock in a child’s residential neighborhood at-risk of presenting with lead-based paint (28.05 vs 24.08, P < 0.001), and neighborhood socioeconomic status (−0.28 vs 0.33, -P < 0.001). Importantly, across every variable for which we observe differences, all function to increase the BLLs of children sampled outside the contraction period relative to children sampled in the PEA traffic contraction period, likely rendering our test results conservative).

#### Atmospheric lead

Finally, we secured data from the Bay Area Air Quality Management District (BAAQMD) measuring atmospheric concentrations of lead at RHV. The BAAQMD data covered the period of 2012 February to 2018 March, with an atmospheric reading taken (on average) every 6 days. We merged these air quality data with our inventory blood lead samples of children ≤5 y of age and residing within 1.5 miles of RHV in the last 10 y.

Given the time-abbreviated nature of the air quality data, only 9,542 of the 14,876 blood lead samples used in our analysis could be assigned an atmospheric lead concentration coincident with the timing of blood draw. The loss of more than 1/3rd of observations warrants some caution in the use of BAAQMD data.

With this caution in mind, for a sampled child *i* in neighborhood block *j* at time *t*, we estimate the responsiveness of child blood lead *Y*_*ijt*_ to atmospheric lead concentration with the following linear model

(8) \begin{eqnarray*} Y_{ijt} &=& \beta _{0} + \beta _{1} D_{it}^{n} + \beta _{2} D_{it}^{f} + \beta _{3} T_{it} + \beta _{4} W_{it}^{e} + \beta _{5} W_{it}^{s} + \beta _{6} W_{it}^{w}\\ && + \beta _{7} PbA_{it} \Gamma _{1} G_{i} + \Gamma _{2} A_{it} + \Gamma _{3} C_{it} + \Gamma _{4} S_{i} + \Gamma _{5} Z_{it} + \Gamma _{6} L_{it}\\ && + \lambda _{1} F_{it} + \lambda _{2} H_{jt} + \lambda _{3} I_{jt} + \lambda _{4} Q_{it} + \gamma _{i} + \gamma _{j} + \varepsilon _{ijt}, \end{eqnarray*}

where, the meaning of all terms carry from Eq. (2), with the exception of *PbA*_*it*_ which captures the 2-month moving average of atmospheric lead (measured in micrograms per cubic meter) from the date of child blood draw. Insofar as exposure to atmospheric lead (measured at RHV) is a source of risk, we expect *β*_7_ to be posi-tive.

## Supplementary Material

pgac285_Supplemental_Files

## Data Availability

The child blood lead data supporting the analysis of this study are available from the Childhood Lead Poisoning Prevention Branch of the California Department of Public Health, were used under license for the current study, and are not publicly available.

## Notes

**Comprting Interests**: The authors declare no competing interests.
