# Supplementary material for: Leaded aviation gasoline exposure risk and child blood lead levels
Source: PNAS Nexus. 2023 Jan 10;2(1):pgac285. doi: 10.1093/pnasnexus/pgac285 (PMC9829455; doi:10.1093/pnasnexus/pgac285)
Supplement: pgac285_Supplemental_Files [file pgac285_supplemental_files.zip › PNASNEXUS-PNASNEXUS-2022-00598-T-s06.pdf]

Supplementary Information for

Leaded Aviation Gasoline Exposure Risk and Child Blood Lead Levels

Sammy Zahran, Christopher Keyes and Bruce Lanphear

Sammy Zahran.
E-mail: szahran@colostate.edu

This PDF file includes:

Figs. S1 to S5
Tables S1 to S6

Table S1. Sample Descriptive Statistics

	Mean	Std. Dev.	Variable	Mean	Std. Dev.
Response Variables			Demographic Variables		
BLL	1.8	1.68	Age (Years)	2.19	1.23
< 1.5	0.436	0.5	Male	0.506	0.50
1.5 to 3	0.436	0.50	Female	0.494	0.50
3 to 4.5	0.092	0.29	Timing Controls		
>4.5	0.015	0.12	2011	0.104	0.31
Exposure Risk Variables			2012	0.097	0.29
Distance to RHV (Miles)	1.02	0.32	2013	0.092	0.28
0-0.5 Miles	0.063	0.24	2014	0.085	0.28
0.5-1 Miles	0.374	0.48	2015	0.116	0.32
1-1.5 Miles	0.563	0.50	2016	0.124	0.33
PEA Traffic (Percentile)	0.51	0.29	2017	0.117	0.33
Tercile (low)	0.34	0.47	2018	0.104	0.31
Tercile (Medium)	0.332	0.47	2019	0.098	0.31
Tercile (High)	0.327	0.47	2020	0.06	0.24
Aviation Gasoline (1,000 Gallon)	23.89	5.7	Spring	0.252	0.43
North	0.343	0.48	Summer	0.268	0.44
East	0.066	0.25	Fall	0.248	0.43
South	0.2	0.4	Winter	0.232	0.422
West	0.39	0.49	Neighborhood SES		
Draw Controls			Median Household Income	68,812	19,834
Non-Capillary Draw	0.748	0.43	Median Home Values	454,227	118,066
Capillary Draw	0.252	0.43	College Educated	13	5.91
Sample Order	0.084	1.03	Other Exposure Sources		
Detected Value	0.556	0.5	Pre-1960 Homes	27.95	21.49
Detection Limited	0.444	0.5	TRI Facilities (<2 Miles)	2.5	0.73

See Table 1 notes from manuscript

Table S2. Coefficients for Various Functions of Residential Distance to Reid-Hillview vis-à-vis Child BLLs

BLLs (μg/dL)	BLL	BLL	BLL	Log BLL	Log BLL	Log BLL
1. Distance RHV (miles)	-0.093**			-0.038***		
	(0.044)			(0.013)		
2. Distance RHV (sqrt miles)		-0.184**			-0.073***	
		(0.081)			(0.024)	
3. Distance RHV (log miles)			-0.086**			-0.033***
			(0.035)			(0.011)
4. PEA Traffic Volume	0.167**	0.167**	0.167**	0.073***	0.073***	0.073***
	(0.065)	(0.065)	(0.065)	(0.015)	(0.015)	(0.015)
5. East Residence	0.244***	0.245***	0.246***	0.148***	0.148***	0.148***
	(0.038)	(0.038)	(0.038)	(0.019)	(0.019)	(0.019)
6. Age (Years)	0.073***	0.073***	0.073***	0.035***	0.035***	0.035***
	(0.013)	(0.013)	(0.013)	(0.003)	(0.003)	(0.003)
7. Female	-0.041	-0.041	-0.041	-0.008	-0.008	-0.008
	(0.027)	(0.027)	(0.027)	(0.006)	(0.006)	(0.006)
8. Capillary Draw	0.611***	0.611***	0.611***	0.392***	0.392***	0.392***
	(0.055)	(0.055)	(0.055)	(0.006)	(0.006)	(0.006)
9. Sample Order	0.134***	0.134***	0.134***	0.020***	0.020***	0.020***
	(0.025)	(0.025)	(0.025)	(0.005)	(0.005)	(0.005)
10. % Before 1960	0.0001	0.0001	0.0001	0.0002	0.0002	0.0002
	(0.0005)	(0.0005)	(0.0005)	(0.0002)	(0.0002	(0.0002)
11. TRI Facilities	0.050***	0.050***	0.050***	0.022***	0.022***	0.022***
	(0.015)	(0.015)	(0.015)	(0.006)	(0.006)	(0.006)
12. Neighborhood SES	-0.066***	-0.066***	-0.066***	-0.031***	-0.031***	-0.031***
	(0.022)	(0.022)	(0.022)	(0.010)	(0.010)	(0.010)
13. Summer	0.081*	0.082*	0.082*	0.036***	0.036***	0.036***
	(0.049)	(0.049)	(0.049)	(0.009)	(0.009)	(0.009)
14. Autumn	0.096***	0.097***	0.097***	0.044***	0.044***	0.045***
	(0.026)	(0.026)	(0.026)	(0.009)	(0.009)	(0.009)
15. Winter	0.026	0.026	0.026	0.008	0.008	0.008
	(0.022)	(0.022)	(0.022)	(0.008)	(0.008)	(0.008)
16. Constant	1.917***	1.999***	1.807***	0.725***	0.757***	0.682***
	(0.302)	(0.301)	(0.309)	(0.096)	(0.097)	(0.097)
Observations	14,804	14,804	14,804	14,804	14,804	14,804
R^2	0.176	0.176	0.176	0.290	0.290	0.290
All Other Controls	Yes	Yes	Yes	Yes	Yes	Yes

Notes: Bootstrapped standard errors in parentheses *** $p < 0.01$, ** $p < 0.05$, * $p < 0.1$; Dependent variable is blood lead level (BLL) in μg/dL and logged BLL where noted; All models limited to children $\leq$ 18 years of age at the time of blood draw, residing $<$ 1.5 miles RHV, and observed from January 1, 2011 to December 31, 2020; Dependent variable in Models (1) to (3) is child BLL (μg/dL); Dependent variable in Models (4) to (6) is the natural log of child BLL (μg/dL); Distances are assigned using the distance (miles) between RHV and the child's place of residence; Residential near angle is defined in equation [1], with east residence representing downwind risk children; PEA traffic is average daily PEA operations at RHV, calculated over 60 days from child's date of draw and normalized. Full saturation of controls includes: child's age (years) and sex (1=female, 0=otherwise), draw method (1=capillary, 0=otherwise), limit of quantification (1=BLL $\leq$ limit of quantification, 0=otherwise), and repeated sample (0=singleton observation, 1,...,n=repeated n times), count of TRI facilities $\leq$ 2 miles from residential address, and percent of neighborhood housing stock built $\leq$ 1960, neighborhood socioeconomic status index, and a set of indicators for season and year-quarter of the date of draw

Table S3. Coefficients for Residential Distance to the Historic San Jose Speedway vis-à-vis Child BLLs

BLLs (μg/dL)	(1)	(2)	(3)	(4)
1. Distance Speedway (miles)		-0.038		-0.091
		(0.043)		(0.075)
2. 0.5 to 1 miles	-0.020		0.006	
	(0.051)		(0.090)	
3. 1 to 1.5 miles	-0.004		0.045	
	(0.044)		(0.095)	
4. >1.5 miles	-0.034		0.007	
	(0.045)		(0.148)	
5. Constant	3.182***	3.198***	2.031***	2.103***
	(0.389)	(0.382)	(0.342)	(0.338)
Observations	14,876	14,876	14,804	14,804
R^2	0.075	0.075	0.077	0.077
RHV Indicators	No	No	Yes	Yes
All Other Controls	Yes	Yes	Yes	Yes

Notes: Bootstrapped standard errors in parentheses *** $p < 0.01$, ** $p < 0.05$, * $p < 0.1$; See Supplemental Table 2 notes.

Table S4. Coefficients for Distance, Near Angle, and PEA Traffic at RHV vis-à-vis Child BLLs under Various Point Locations to the Airport

BLLs (μg/dL)	(1)	(2)	(3)	(4)	(5)
1. Distance RHV (miles)	-0.105**				
	(0.047)				
2. Distance RHV, Point A (miles)		-0.113**			
		(0.051)			
3. Distance RHV, Point B (miles)			-0.112**		
			(0.052)		
4. Distance RHV, Point C (miles)				-0.103**	
				(0.050)	
5. Distance RHV, Point D (miles)					-0.105*
					(0.059)
6. PEA Traffic	0.201***	0.200***	0.198***	0.203***	0.207***
	(0.066)	(0.067)	(0.067)	(0.067)	(0.066)
7. East	0.270***	0.324***	0.361***	0.268***	0.218***
	(0.042)	(0.049)	(0.051)	(0.049)	(0.059)
8. Constant	1.978***	1.889***	1.836***	2.018***	2.101***
	(0.316)	(0.313)	(0.327)	(0.323)	(0.302)
All Other Controls	Yes	Yes	Yes	Yes	Yes

Notes: Bootstrapped standard errors in parentheses *** $p < 0.01$, ** $p < 0.05$, * $p < 0.1$; Point coordinates (Latitude, Longitude) for RHV distance calculations: (37.3362252, -121.8230194); Point location coordinates for alternative distance calculation robustness tests: Point A: (37.333420, -121.820492), Point B: (37.332981, -121.819312), Point C: (37.331898, -121.818239), Point D: (37.329748, -121.816147); See Supplemental Table 2 notes.

Table S5. Coefficients for Distance, Near Angle, and PEA Traffic at RHV vis-à-vis Child BLLs under Various Single Imputation Operations for Detection Limited Observations

BLLs (μg/dL)	(1)	(2)	(3)
1. Distance RHV (0.5-1 miles)	-0.221**	-0.220**	-0.055***
	(0.066)	(0.065)	(0.018)
2. Distance RHV (1-1.5 miles)	-0.218***	-0.217***	-0.058***
	(0.064)	(0.064)	(0.015)
3. PEA Traffic Volume	0.168***	0.168***	0.073***
	(0.064)	(0.064)	(0.015)
4. East Residence	0.162***	0.159***	0.144***
	(0.032)	(0.032)	(0.010)
5. Constant	2.161***	2.167***	0.746***
	(0.318)	(0.318)	(0.097)
Observations	14,804	14,804	14,804
All Other Controls	Yes	Yes	Yes
BLL/$\sqrt{2}$	Yes	No	No
BLL$\times\log 2$	No	Yes	No
$\ln$(BLL/$\sqrt{2}$	No	No	Yes

Notes: Bootstrapped standard errors in parentheses *** $p < 0.01$, ** $p < 0.05$, * $p < 0.1$; See Supplemental Table 2 notes. We capitulate a series of standard single imputation operations for test results at or below the limit of quantification, including: 1) BLL/$\sqrt{2}$; 2) BLL$\times\log 2$; and $\ln(\text{BLL}/\sqrt{2})$

Table S6. Coefficients for PEA Traffic Terciles at RHV vis-à-vis Child BLLs

BLLs (μg/dL)	BLL	Log BLL
1. PEA Traffic Tercile II	0.097***	0.039***
	(0.039)	(0.001)
2. PEA Traffic Tercile III	0.140***	0.56***
	(0.048)	(0.012)
3. Constant	2.033***	0.762***
	(0.418)	(0.103)
Observations	14,804	14,804
All Other Controls	Yes	Yes

Notes: Bootstrapped standard errors in parentheses *** $p < 0.01$, ** $p < 0.05$, * $p < 0.1$; See Supplemental Table 2 notes.

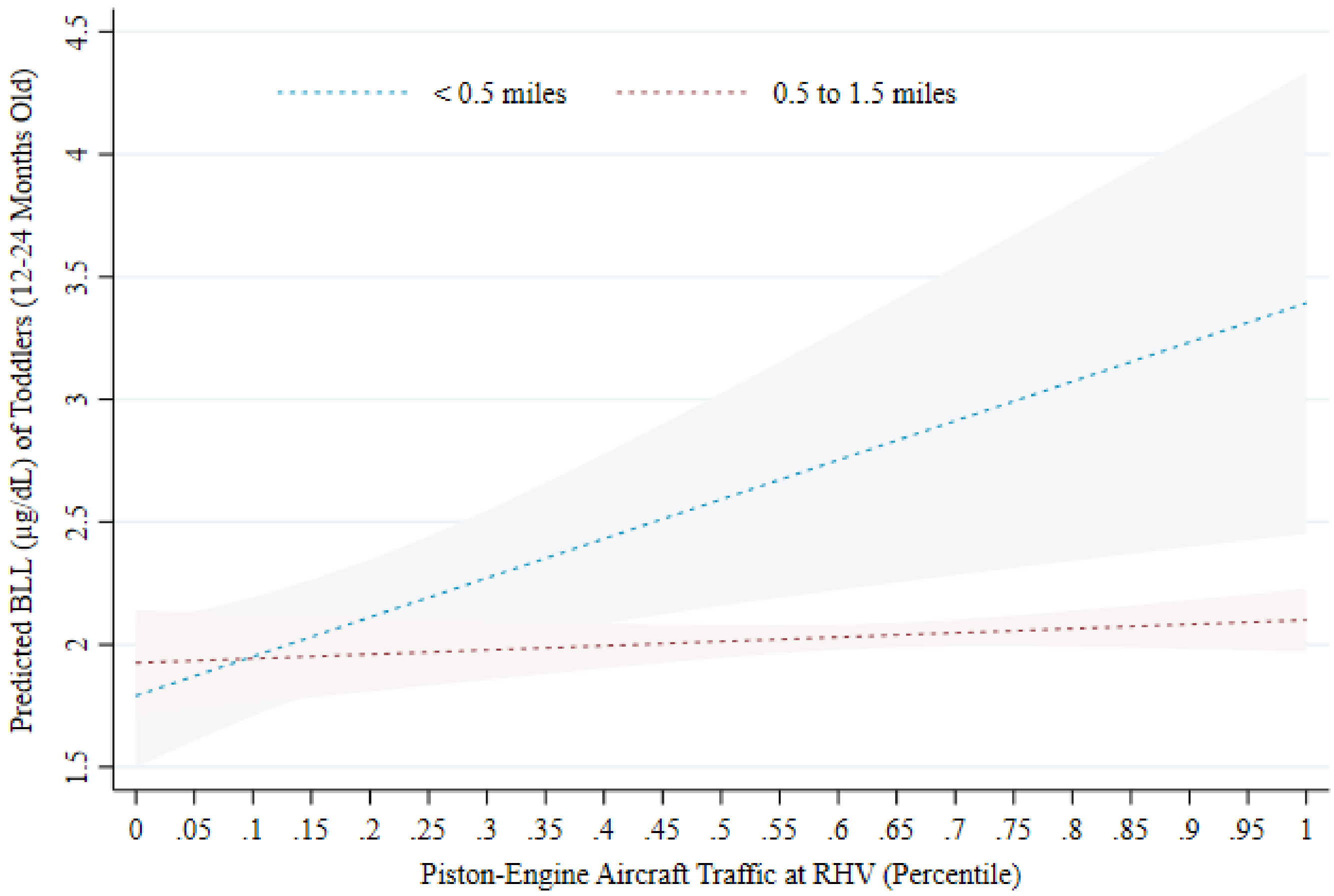

Fig. S1. Predicted Child BLLs for Toddlers (12-24 Months) by Residential Distance and PEA Traffic.

Estimates are from Equation 2. Predicted values are derived by fixing all other model covariates at their sample means.

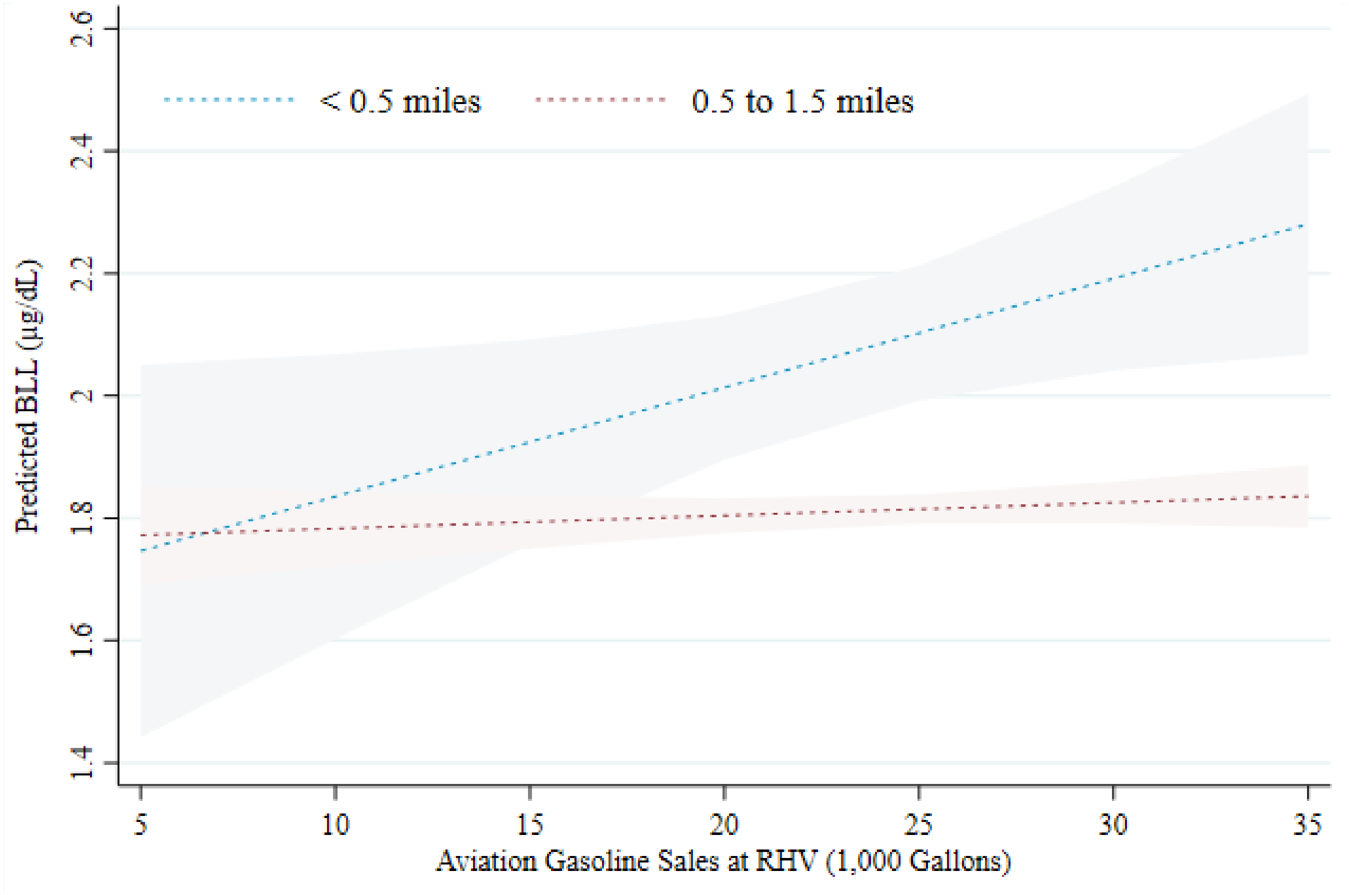

Fig. S2. Predicted Child BLLs by Residential Distance and Quantity of Aviation Gasoline Sold.

Estimates are from Equation 2 where the piston-engine aircraft traffic variable is substituted for the quantity of aviation gasoline sold. Predicted values are derived by fixing all other model covariates at their sample means.

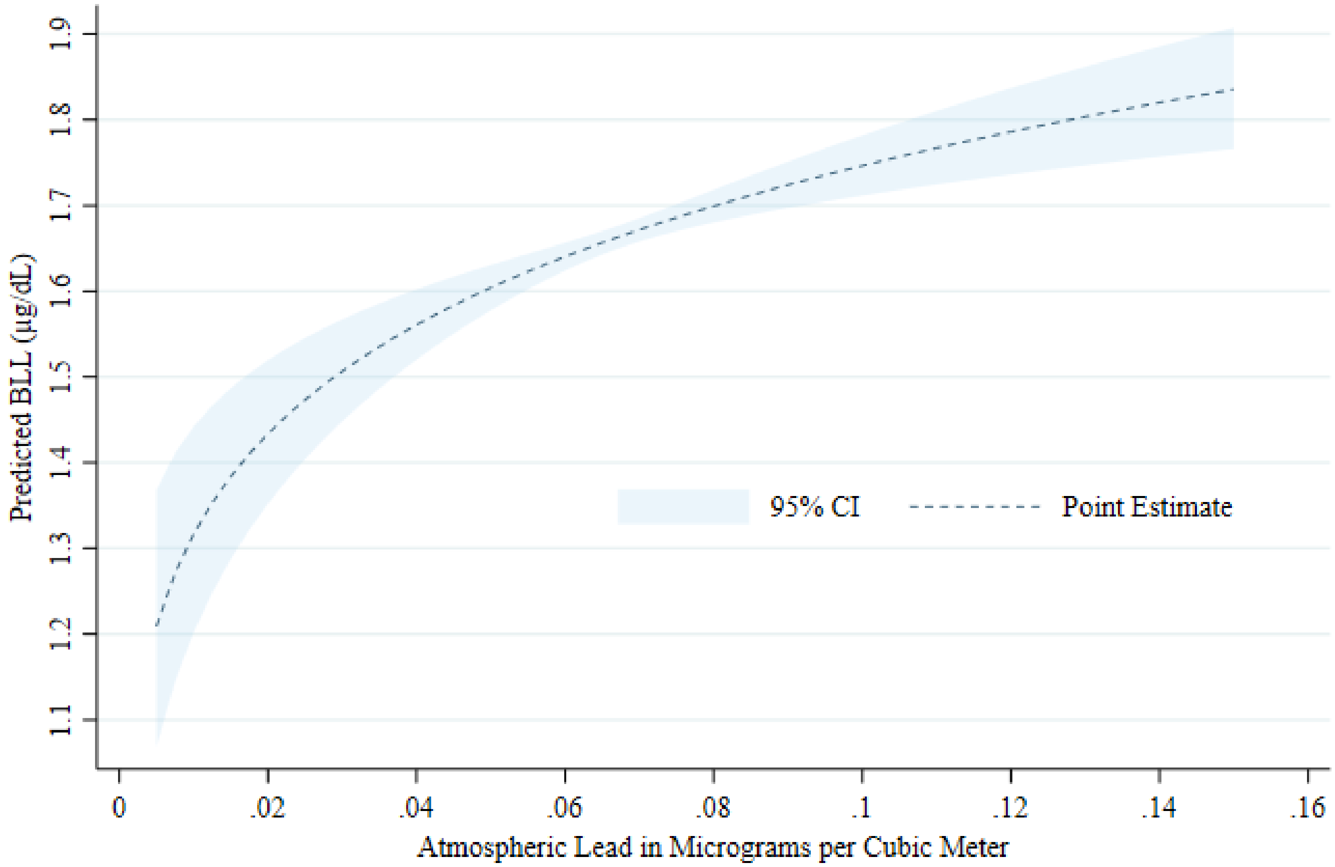

Fig. S3. Predicted Child BLLs by Atmospheric Concentration of Lead.

Estimates are from Equation 8. Predicted values are derived by fixing all other model covariates at their sample means.

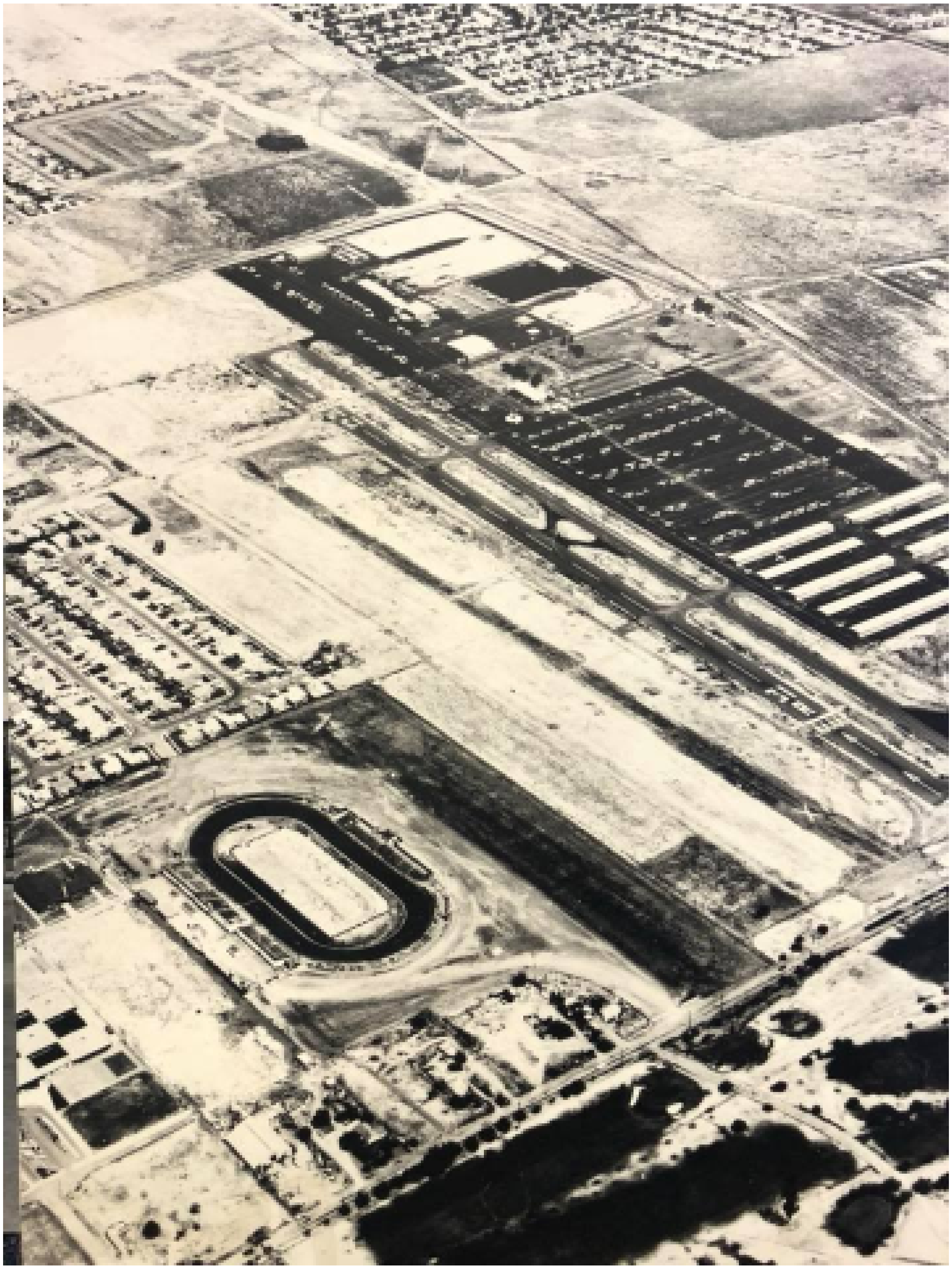

Fig. S4. Aerial Photograph of the San Jose Speedway

We calculated the Haversine distance from a sampled child's residence to the historic location of the San Jose Speedway (point coordinates 37.3293856, -121.8202305). Aerial photograph was provided by Michael McDonald from http://www.legendsofsanjosespeedway.com/

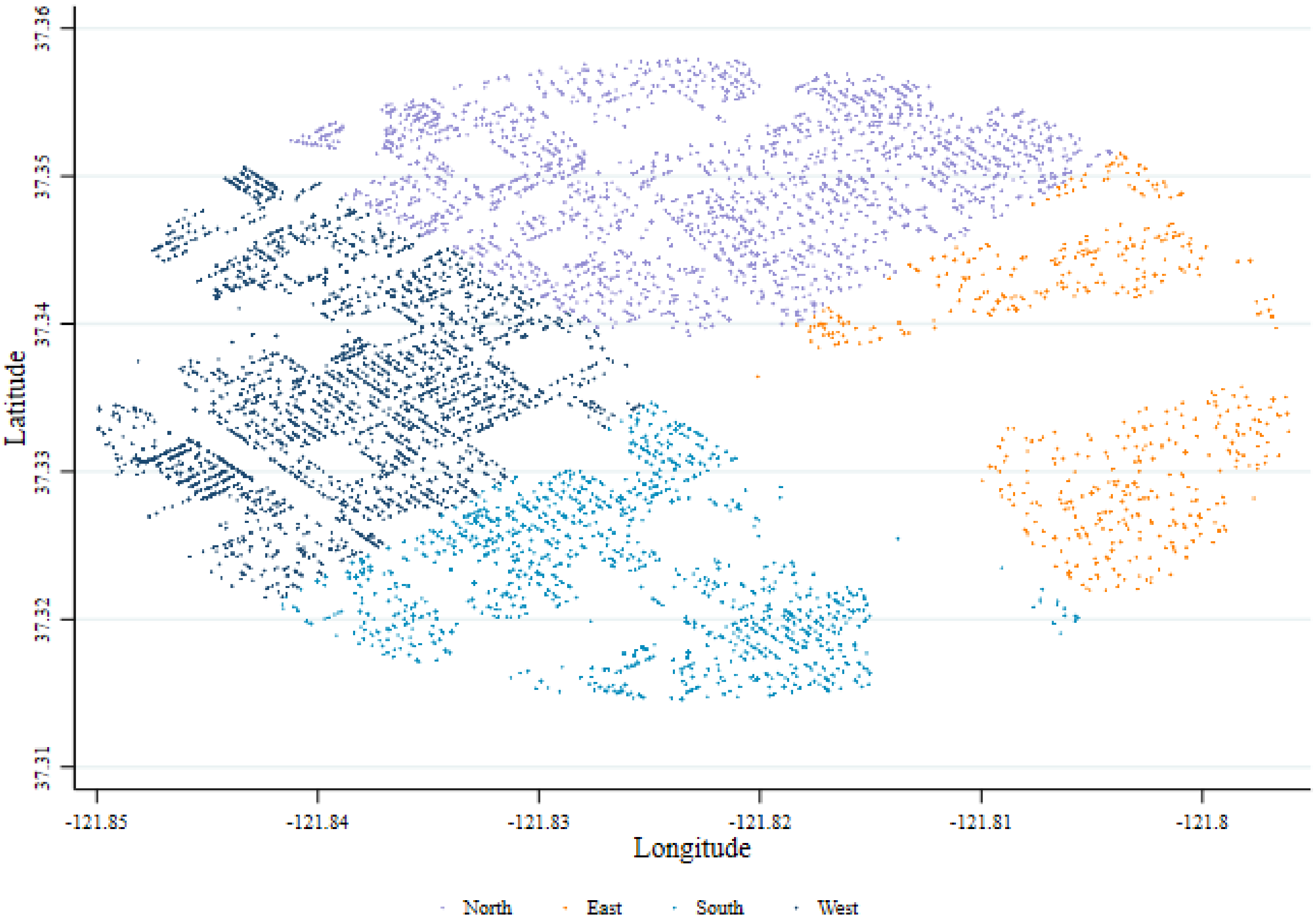

Fig. S5. Spatial Distribution of Sampled Children by Near Angle Groupings

A near angle group was assigned to each address by calculating the compass bearing (degrees) between a child's residential location and RHV. We define near angle groups by the four cardinal directions: North (N), East (E), South (S) and West (W).
